# Two well-differentiated pancreatic neuroendocrine tumor mouse models

**DOI:** 10.1038/s41418-019-0355-0

**Published:** 2019-06-03

**Authors:** Chung Wong, Laura H. Tang, Christian Davidson, Evan Vosburgh, Wenjin Chen, David J. Foran, Daniel A. Notterman, Arnold J. Levine, Eugenia Y. Xu

**Affiliations:** 1 Raymond and Beverly Sackler Foundation Laboratory, New Brunswick, NJ 08901 USA; 2 Department of Pathology, Memorial Sloan-Kettering Cancer Center, New York, NY 10065 USA; 3 Department of Pathology, University of Utah, Huntsman Cancer Institute, Salt Lake City, UT 84112 USA; 4 Rutgers Cancer Institute of New Jersey, Rutgers, the State University of New Jersey, New Brunswick, NJ 08903 USA; 5 Department of Medicine, Robert Wood Johnson Medical School, Rutgers, the State University of New Jersey, New Brunswick, NJ 08901 USA; 6 Department of Pathology and Laboratory Medicine, Robert Wood Johnson Medical School, Rutgers, the State University of New Jersey, New Brunswick, NJ 08901 USA; 7 Department of Molecular Biology, Princeton University, Princeton, NJ 08544 USA; 8 School of Natural Sciences, Institute for Advanced Study, Princeton, NJ 08540 USA; 9 Department of Pediatrics, Robert Wood Johnson Medical School, Rutgers, the State University of New Jersey, New Brunswick, NJ 08901 USA; 10 Present Address: Department of Molecular Biology, Princeton University, Princeton, NJ 08544 USA; 11 Present Address: Regeneron Inc., Tarrytown, NY 10591 USA; 12 Department of Medicine, Yale University School of Medicine, New Haven, CT 06510 USA

**Keywords:** Cancer models, Tumour-suppressor proteins

## Abstract

Multiple endocrine neoplasia type 1 (MEN1) is a genetic syndrome in which patients develop neuroendocrine tumors (NETs), including pancreatic neuroendocrine tumors (PanNETs). The prolonged latency of tumor development in MEN1 patients suggests a likelihood that other mutations cooperate with *Men1* to induce PanNETs. We propose that *Pten* loss combined with *Men1* loss accelerates tumorigenesis. To test this, we developed two genetically engineered mouse models (GEMMs)—MPR (*Men1*^*flox/flox*^ *Pten*^*flox/flox*^ RIP-Cre) and MPM (*Men1*^*flox/flox*^ *Pten*^*flox/flox*^ MIP-Cre) using the Cre-LoxP system with insulin-specific biallelic inactivation of *Men1* and *Pten*. Cre in the MPR mouse model was driven by the transgenic rat insulin 2 promoter while in the MPM mouse model was driven by the knock-in mouse insulin 1 promoter. Both mouse models developed well-differentiated (WD) G1/G2 PanNETs at a much shorter latency than *Men1* or *Pten* single deletion alone and exhibited histopathology of human MEN1-like tumor. The MPR model, additionally, developed pituitary neuroendocrine tumors (PitNETs) in the same mouse at a much shorter latency than *Men1* or *Pten* single deletion alone as well. Our data also demonstrate that Pten plays a role in NE tumorigenesis in pancreas and pituitary. Treatment with the mTOR inhibitor rapamycin delayed the growth of PanNETs in both MPR and MPM mice, as well as the growth of PitNETs, resulting in prolonged survival in MPR mice. Our MPR and MPM mouse models are the first to underscore the cooperative roles of *Men1* and *Pten* in cancer, particularly neuroendocrine cancer. The early onset of WD PanNETs mimicking the human counterpart in MPR and MPM mice at 7 weeks provides an effective platform for evaluating therapeutic opportunities for NETs through targeting the MENIN-mediated and PI3K/AKT/mTOR signaling pathways.

## Introduction

Neuroendocrine tumors (NETs) constitute a heterogeneous group of neoplasms that can arise from the NE cells found in numerous tissues throughout the body including the gastroenteropancreatic tract, bronchopulmonary system, pituitary, parathyroids, thyroid, and ovaries. Pancreatic NETs (PanNETs) are found in the gastroenteropancreatic tract. Human pancreatic NE neoplasms are classified as either well-differentiated (WD) tumor (WD-NET) or poorly differentiated (PD) carcinoma (PD-NEC) [1]. WD PanNETs can be functional, secreting biologically active hormones such as insulin, glucagon, and others, or non-functional. PD-PanNECs are genetically and biologically related to conventional carcinoma with worse clinical prognosis [2]. Based on Ki 67 index, WD PanNETs are graded as G1 (<3%), G2 (3–20%), or G3 (>20%) [3, 4].

When surgery is not an option, the Food and Drug Administration has three approved drugs to treat progressive PanNETs: everolimus (rapamycin analog), sunitinib, and radiotherapy Lutathera (somatostatin analogs) [5–8]. The preclinical efficacy of rapamycin and sunitinib was demonstrated using the human BON-1 xenograft and RIP-Tag2 mouse models [9–13]. These mice develop PanNETs with poorly differentiated and high-grade histology, which do not resemble the counterpart of human PanNETs [14, 15]. Additional preclinical murine models that more closely reflect the histology and behavior of human WD PanNETs are needed.

Multiple endocrine neoplasia type 1 (MEN1) is an autosomal-dominant inherited syndrome with manifestation of NETs that involve at least two of the four endocrine glands, frequently parathyroid glands, endocrine pancreas, anterior pituitary, and adrenal gland [16–19]. The *MEN1* gene is responsible for the syndrome. Its gene product, MENIN, is a highly conserved tumor suppressor [20]. Biallelic inactivation of *MEN1* occurs in 44% of human PanNETs, inherited or sporadic, and is sufficient to drive tumorigenesis with long latency [21]. The delayed latency of NET development suggests that additional molecular and genetic events might be required for tumorigenesis.

The human and mouse genes share a highly conserved genomic structure with 89% nucleotide sequence homology and 97% amino acid sequence homology, respectively [22]. Mouse strains with defective *Men1* possess remarkable phenotypic and histological overlap with the human *MEN1* syndrome. Heterozygous *Men1* mice or homozygous β-cell-specific *Men1* deletion mice develop WD PanNETs and pituitary neuroendocrine tumors (PitNETs) also with long latency [23–28] as human MEN1 patients.

The tumorigenic latency in the Men1 mouse model makes it less ideal for the preclinical testing of candidate drugs. Identifying genes that function cooperatively with *Men1* could help us develop a better preclinical WD PanNET mouse model. In seeking targets, we consider the phosphoinositide 3-kinase (PI3K)/protein kinase B (AKT)/mammalian target of rapamycin (mTOR) signaling pathway, the second most mutated pathway in cancer, after p53 [29]. The mTOR pathway plays an important role in human NETs based on genome sequencing [21, 30–34]. Additionally, an mTOR inhibitor, everolimus, is used to treat PanNET patients. Phosphatase and tensin homolog (PTEN), a key negative regulator of the PI3K/AKT/mTOR pathway, is frequently mutated or lost in several familial or sporadic cancer types; however, in PanNETs, the frequency of loss is low, 7–26.4% [21, 32–38]. Co-mutations of *MEN1* and *PTEN* have been observed in a small percentage of human PanNETs [21, 32]. Thus we hypothesize that Menin and Pten may function cooperatively to suppress NE tumorigenesis.

Here we generated two genetically engineered mouse models (GEMMs) harboring homozygous deletions of *Men1* and *Pten* within insulin-producing β-cells and compared histopathology with the Men1 and Pten mouse models. Concomitant loss of *Pten* and *Men1* accelerated NE tumorigenesis. These GEMMs could provide improved preclinical therapeutic models for WD PanNET.

## Methods and materials

### Animals

To generate compound mice *Men1*^*flox/flox*^ *Pten*^*flox/flox*^ RIP-Cre (MPR) (Supplementary Fig. S1A), *Men1*^*flox/flox*^ mice (129S(FVB)-*Men1*^*tm1.2Ctre*^/J, stock number 005109, The Jackson Laboratory, USA) were first crossed with *Pten*^*flox/flox*^ mice (C;129S4-*Pten*^*tm1Hwu*^/J, stock number: 004597, The Jackson Laboratory, USA) to generate heterozygous *Men1*^*+/flox*^ *Pten*^*+/flox*^ mice. The resulting mice were then intercrossed to generate *Men1*^*flox/flox*^ *Pten*^*flox/flox*^ (MP) mice. The resulting homozygous mice were then crossed with RIP-Cre mice (C57BL/6-Tg(Ins2-cre) 25Mgn/J, stock number: 003573, The Jackson Laboratory, USA) to generate *Men1*^*+/flox*^ *Pten*^*+/flox*^ RIP-Cre mice. These mice were further crossed back to MP mice to generate the desired MPR compound mice and corresponding littermates MP. Confirmation of the genotypes in mice was evaluated by PCR using tail genomic DNA (Supplementary Fig. S1B). Tissue-specific deletion of *Men1* and/or *Pten* genes was confirmed by PCR using genomic DNA from various organs. Supplementary Fig. S1C showed that *Men1* and *Pten* genes were specifically deleted in pancreatic islets and brain, but not in heart, intestine, kidney, liver, lung, spleen, and pancreatic exocrine tissues in the representative MPR mice. This is consistent with the previous report that RIP-Cre is specifically expressed in pancreatic islets and hypothalamus [24, 39].

*Pten*^*flox/flox*^ RIP-Cre (PR) mice were produced by generating heterozygous *Pten*^*+/flox*^ RIP-Cre animals by the first cross of *Pten*^*flox/flox*^ mice with RIP-Cre mice, then by crossing the resulting *Pten*^*+/flox*^ RIP-Cre mice with *Pten*^*flox/flox*^ mice. *Men1*^*flox/flox*^ RIP-Cre (MR) mice were produced similarly as the strategy to produce PR mice. The same strategy was taken to generate compound mice *Men1*^*flox/flox*^ *Pten*^*flox/flox*^ MIP-Cre (MPM), *Men1*^*flox/flox*^ MIP-Cre (MM), and *Pten*^*flox/flox*^ MIP-Cre (PM), except that MIP-Cre (B6(Cg)-*Ins1*^*tm1.1(cre)Thor*^/J, The Jackson Laboratory, USA) mice were used instead of RIP-Cre mice. To generate MPM, MM, and PM mice more quickly, first-generation MPM, MM, or PM mice were bred with MP, *Men1*^*flox/flox*^, or *Pten*^*flox/flox*^ mice, respectively, to obtain second-generation MPM, MM, or PM mice for the experiments. Animals were genotyped by using vendors’ recommended primers (The Jackson Laboratory, USA) [40] and standard genomic PCR techniques. All cohorts were in a mixed genetic background. Animals were housed in a temperature-, humidity-, and light-controlled room (12-h light/dark cycle), allowing free access to food and water.

Mice were studied alongside age- and sex-matched control animals unless otherwise indicated.

All animal experiments were conducted according to the research guidelines set forth by the Institutional Animal Care and Use Committee (IACUC) of Rutgers, the State University of New Jersey, USA.

### Evaluation of pituitary size

A ruler was used to measure the size of a pituitary at autopsy. The volume of a pituitary was calculated with the formula *V* = (π/6) × (length × width × height). The dimensions of a normal pituitary are: 3–3.5 mm in length, 1–1.5 mm in width and depth.

### Evaluation of PanNET formation

To score PanNETs, three 120-μm apart pancreas sections from each mouse were stained with hematoxylin and eosin (H & E), glucagon, and insulin. The sections were evaluated histologically. One or more islets of ≥1 mm in diameter with loss of α-cells (negative immunoreactivity for glucagon) and clonal proliferation of β-cells (positive immunoreactivity for insulin) in any of the three sections were considered as tumor development (PanNET) in that mouse.

### Histology and immunohistochemistry (IHC)

Tissues were fixed in 10% buffered formalin solution (Fisher Scientific, Inc., USA) for 24 h at room temperature or for 48 h at 4 °C. Fixed tissues were then washed in 50% ethanol and transferred to 70% ethanol for paraffin embedding. For IHC, paraffin-embedded tissues were cut into 4-μm sections and stained with H&E. All IHC staining was performed on 4-μm paraffin-embedded sections and placed on charged glass slides. Sections were de-waxed with histoclear (National Diagnostics, Inc., USA) and rehydrated through graded alcohol. Antigen retrieval was then performed by incubating the slides in antigen retrieval solution (Vector Labs, USA) at 95 °C for 15 min. Slides were then allowed to cool for 20 min on ice. After washing in phosphate-buffered saline (PBS) with 0.1% Tween 20, the slides were blocked with a 3% hydrogen peroxide solution for 10 min. The slides were then washed in PBS with 0.1% Tween 20. The endogenous biotin activity was inactivated using the Endogenous Biotin Blocking Kit (Invitrogen, Inc., USA). The following detection and visualization procedures were performed according to the manufacturers’ protocol. Slides were counterstained in Gill’s hematoxylin, dehydrated, cleared, and cover-slipped. Negative control slides were run without primary antibody. Control slides known to be positive for each antibody were incorporated. DAKO antibodies (Fisher Scientific, USA): Insulin (A0564), Prolactin (A0569), and Growth hormone (GH) (A0570); Cell Signaling antibodies (Cell Signaling Technology Inc., Danvers, MA, USA): Glucagon (2760), Pten (9559); Abcam antibodies: Chromograinin A (ab15160), Ki 67 (ab15580), adrenocorticotropin hormone (ACTH) (ab74967); synaptophysin (Roche, 790-4407, USA); and Menin (Bethyl Lab, a300-105a) were used.

### Proliferation index

For quantification of IHC positive staining for Ki 67, the areas with the highest density of Ki 67 reactivity among tumor cells were first identified. At least 1000 cells were counted at ×20 magnification in these high Ki 67 density areas in a minimum of three mice of each genotype and sex.

### Ratio of the islets area per pancreas area

IHC-insulin-stained pancreas sections from three 120-μm apart sections per mouse (three or more samples of each genotype and sex were used) were digitized at ×20 at Rutgers Cancer Institute of New Jersey Biomedical Informatics shared resource using an Olympus VS120 whole slide scanner (Olympus Corporation of the Americas, Center Valley, PA). The image analysis algorithm was custom developed on Visiopharm image analysis platform (Visiopharm A/S, Hoersholm, Denmark). Insulin-positive pancreatic islets were digitally recognized and outlined. The diameter and area of individual islets were measured accordingly, and the area of the whole pancreas was measured as well. For quantitative analysis, the islets-to-pancreas ratio was calculated and graphed.

### Molecular analysis

Genomic DNA was isolated using the QIAamp DNA Mini Kit (Qiagen, USA) and total RNA was isolated using the RNeasy Mini Kit (Qiagen, USA) per the manufacturer’s instruction. PCR fragments from genomic DNA were amplified using a thermal cycler (Veriti, the Applied Biosystems, USA) (94 °C, 3 min; 94 °C, 30 s, 60 °C, 1 min, 72 °C, 1 min, for 40 cycles; 72 °C, 7 min). cDNA was synthesized from 1 μg of total RNA using TaqMan® Reverse Transcription Reagents (Life Technologies, Grand Island, NY, USA) per the manufacturer’s instruction. Real-time PCR was performed as described before [41]. All experiments were performed in triplicate and each experiment was repeated at least twice independently.

### Western blot analysis

Protein lysates were made from tissues and tumor samples using RIPA lysis buffer (ThermoFisher Inc., USA) containing the complete protease inhibitor cocktail (Roche, USA) and the PhosSTOP phosphatase inhibitor cocktail (Roche, USA). Ten μg of protein lysate was loaded onto sodium dodecyl sulfate-polyacrylamide gel electrophoresis gels and transferred to polyvinylidene difluoride membrane for immunoblotting as described previously [41]. Membranes were probed with antibodies. The following antibodies were purchased from Cell Signaling Technology Inc. (Danvers, MA, USA): AKT, p-AKT (S473), p-RPS6 (S235/236), RPS6, PTEN. Antibody against Menin was purchased from Bethyl Laboratories, Inc. (Montgomery, TX, USA). GAPDH protein was used as a loading control for immunoblots in all the experiments. All the experiments were repeated at least twice independently.

### Serum assays

Since MPR mice showed lethargic symptom after 9 weeks, to make sure MPR mice were alive for glucose measurement and blood collection for serum assays, all MPR, control MP littermates, MR, and PR mice were fasted for 3–5 h in the morning before blood collection. MPM and control MP littermates were fasted for 16 h before blood collection. Blood glucose was measured with ONE TOUCH Ultra2 blood glucose meter (Lifescan, Inc., USA). Using the manufacturer’s instructions, serum insulin levels were determined with an ultrasensitive mouse insulin ELISA Kit (Crystal Chem Inc., 90080, USA). Serum prolactin, GH, and ACTH levels were measured using commercial kits from Calbiotech (PR063F-100, USA), Millipore (EZRMGH-45K, USA), and Lifespan Biosciences Inc. (LS-F5354, USA), respectively. Serum insulin, prolactin, and GH levels were repeated at least twice independently. Serum ACTH levels was performed once due to limitation of serum.

### Rapamycin treatments

The in vivo efficacy of rapamycin treatment on NETs was evaluated in MPR mouse model. One trial was on 4–5-week-old MPR mice and the other was on 7–9-week-old mice. Vehicle and rapamycin (LC Laboratories Inc., Woburn, MA, USA) (15 mg/kg, QWK, I.P.) was injected into mice weekly. Body weight was measured once per week. Pituitary size was measured at autopsy using a ruler. Pituitary and pancreas were examined macroscopically at autopsy. Paraffin-embedded sections were evaluated histologically after H & E and IHC staining.

The in vivo efficacy of rapamycin was similarly evaluated in the MPM mouse model. Treatments were performed on 4-week-old MPM mice. Pancreas was examined at autopsy macroscopically. Paraffin-embedded sections were evaluated histologically after H&E and IHC staining.

### Statistical analysis

Graphs were produced using the GraphPad Prism version 6.0b software. The statistical significance of survival curves between two groups was analyzed using log-rank (Mantel-Cox) test, and the statistical significance of pituitary size between two groups was analyzed using unpaired *t* test with Welch’s correction by the GraphPad Prism_6.0b software. *p* < 0.05 was considered significant.

## Results

### *Pten* and *Men1* function cooperatively to accelerate PitNETs and death

**Fig. 1:**
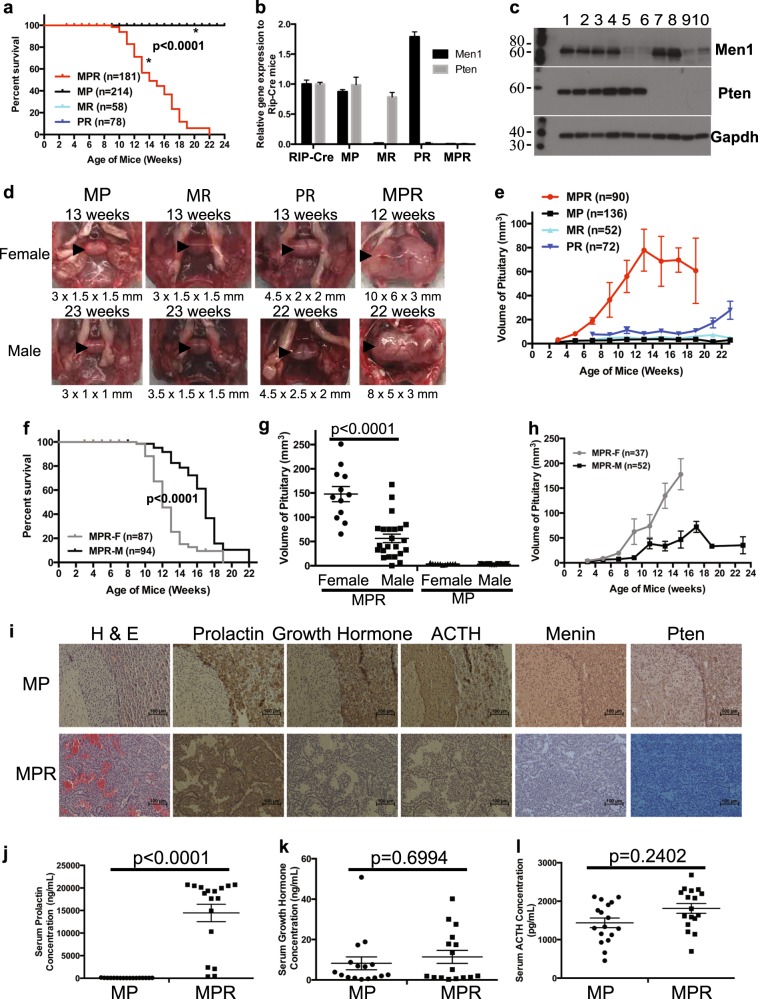
Concomitant loss of *Men1* and *Pten* decreased survival and accelerated pituitary neuroendocrine tumor (PitNET) development in MPR mice. **a** Kaplan–Meier survival curves showed significantly shorter life span (*p* < 0.0001) in MPR mice than in MR, PR, and control MP mice. **b**, **c** Quantitative mRNA and western blots showed that corresponding Men1 or/and Pten gene/protein was deleted in pituitary in the representative mice of various genotypes. **b** Quantitative mRNA analysis. **c** Western blots analysis. Genotypes of each lane: 1 and 2—RIP-Cre; 3 and 4—MP; 5 and 6—MR; 7 and 8—PR; 9 and 10—MPR. The molecular weight markers (in kD) are labeled on the left side of the blots. **d** Gross pathology of pituitary is shown from representative MP, MR, PR, and MPR female mice at 12–13 weeks and male mice at 22–23 weeks. Normal pituitary is cylindrical in shape. The size of pituitary under the image was written  as length × width × height (mm). Pituitary is shown with arrowhead inside the mouse skull. **e** Evaluation of the size of pituitaries in 2-week interval starting at 3 weeks (MPR and MP mice) or 7 weeks (MR and PR mice) showed that PitNETs developed dramatically faster and larger in MPR mice. **f** Kaplan–Meier survival curves demonstrated that female MPR mice had significantly shorter life span (*p* < 0.0001) than male MPR mice. **g** Size of pituitary at death in female (*n* = 12) and male (*n* = 23) MPR mice, as well as age-matched female (*n* = 11) and male (*n* = 23) MP mice. **h** Evaluation of the size of pituitaries in female and male MPR mice at scheduled autopsy (*n* = 37 for female mice with *n* = 4, 6, 7, 7, 4, 7, 2 at 3, 5, 7, 9, 11, 13, 15 weeks, respectively; *n* = 52 for male mice with *n* = 3, 4, 3, 7, 4, 9, 10, 9, 1, 0, 2 at 3, 5, 7, 9, 11, 13, 15. 17, 19, 21, 23 weeks respectively). **i** Immunohistochemical staining of prolactin, growth hormones, adrenocorticotropin hormone (ACTH), and Men1 and Pten on pituitary sections in MP and MPR mice. **j**–**l** Serum hormone levels in MP and MPR mice confirmed that these PitNETs were prolactinomas. **j** Prolactin; **k** Growth hormone; **l** ACTH. MPR *Men1*^*flox/flox*^ *Pten*^*flox/flox*^ RIP-Cre, MP *Men1*^*flox/flox*^ *Pten*^*flox/flox*^, MR *Men1*^*flox/flox*^ RIP-Cre, PR *Pten*^*flox/flox*^ RIP-Cre

To test our hypothesis that Pten and Menin may function cooperatively to suppress NE tumorigenesis, we investigated whether the MPR compound mice developed NETs earlier than MR or PR mice. We monitored survival of a cohort of MPR mice, alongside MP mice, the single MR or PR deletion mice. MPR mice started dying at 9 weeks and did not live beyond 23 weeks (Fig. 1a), while control MP, MR, and PR mice did not die during the study period, consistent with other studies [25, 42]. Quantitative mRNA and western blot analysis confirmed that Men1 and/or Pten expression was knocked down in the pituitary in representative MPR, MR, and PR mice (Fig. 1b, c). The median survival of MPR mice was 14 weeks. Among the 70 (38F/32M) lethargic MPR mice, 58 mice (83%, 35F/23M) showed symptoms such as blindness, tilted head/body, circular gait path, and hind legs paralysis—symptoms consistent with those described in human patients with PitNETs. Autopsy of lethargic mice revealed PitNETs and dramatically enlarged pituitaries. Control MP, MR, and PR mice displayed normal or only slightly enlarged pituitaries (Fig. 1d). Evaluation of the pituitary size over time in a cohort of MPR, PR, MR, and MP mice showed that pituitaries grew dramatically faster and larger in MPR mice (Fig. 1e). PR mice showed slightly faster and bigger pituitaries than that in MR and control MP mice during the study period, suggesting that Pten plays a role in suppressing pituitary tumorigenesis. Concomitant loss of *Pten* and *Men1* in mice resulted in earlier onset of PitNETs and death compared to MR and PR mice.

Consistent with the human MEN1 syndrome [43], MPR mice recapitulated a gender bias in tumor development. Assessment of Kaplan–Meier survival (KMS) curves confirmed shortened survival in female vs. male MPR mice (Fig. 1f). Median survival was 12 weeks for females and 17 weeks for males. The PitNETs in female lethargic MPR mice were more than two-fold larger than that in male ones at death, while control female and male MP mice had the same normal size of pituitaries (Fig. 1g). Evaluation of the pituitary size over time in a cohort of MPR mice showed that pituitaries grew faster and larger in female than in male MPR mice (Fig. 1h). This gender bias has also been reported in MR mice [25] and observed in PR mice (Fig. 1d).

To understand the pituitary origin of these tumors, the PitNETs from lethargic MPR mice (*n* = 26, 14F/12M) and the pituitaries from control MP mice were IHC stained for prolactin, GH, and ACTH. Control MP mice showed staining consistent with a normal pituitary—heterogeneous expression of prolactin, GH, and ACTH in the anterior lobe, positive expression of ACTH in the intermediate lobe, and no expression of prolactin, GH, and ACTH in the posterior lobe (Fig. 1i and Supplementary Fig. S2). PitNETs from both female and male MPR mice showed positive staining of prolactin and negative staining of GH and ACTH (Fig. 1i). Evaluation of serum prolactin, GH, and ACTH levels confirmed that these PitNETs were prolactinomas (Fig. 1j–l). Thus these PitNETs arose from the *pars distalis*, mimicking the human MEN1-like syndrome in which prolactinomas are the most common pituitary lesions.

### *Pten* and *Men1* function cooperatively to accelerate PanNETs

**Fig. 2:**
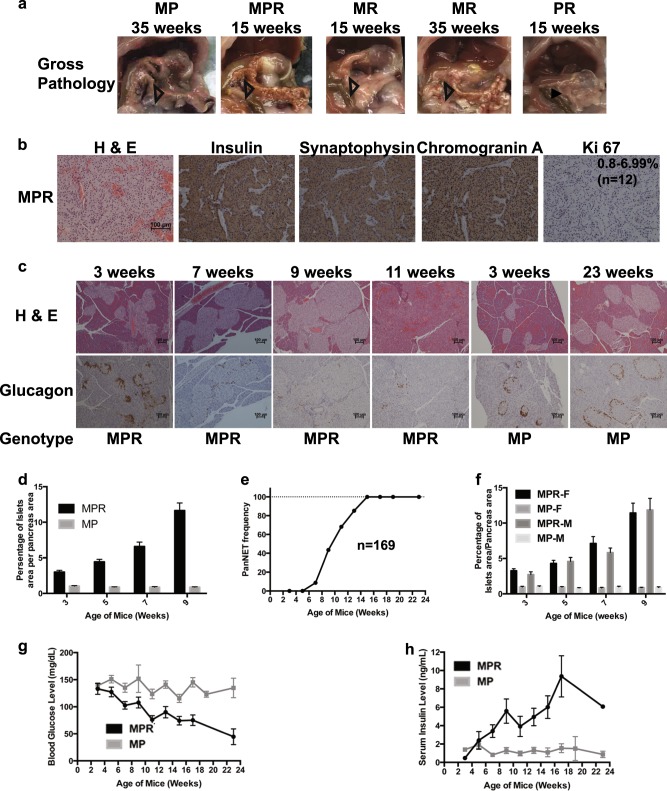
Concomitant loss of *Men1* and *Pten* accelerated pancreatic neuroendocrine tumor (PanNET) development in MPR mice. **a** Gross pathology of pancreas in MP, MPR, MR, and PR mice at 15 or 35 weeks. Pancreas is shown with open triangle inside the mouse abdomen. **b** Hematoxylin and eosin (H & E) and immunohistochemical (IHC) staining of insulin and NET markers on MPR pancreatic tumors. Ki 67 index is shown. **c** H & E and IHC staining of glucagon of pancreas sections from MPR and MP mice of different ages. **d** Quantitative comparison of the ratio of the islets area per pancreas area over time in MP and MPR mice (*n* ≥ 10 at each time point). **e** Frequency of PanNETs in MPR mice at the scheduled autopsy (*n* = 169 in total, *n* = 12, 18, 23, 23, 22, 27, 23, 17, 2, 0, 2 at 3, 5, 7, 9, 11, 13, 15, 17, 19, 21 and 23 weeks, respectively). **f** Quantitative comparison of the ratio of the islets area per pancreas area over time in MPR female and male mice (*n* ≥ 5 at each time point of each genotype and sex). **g** Blood glucose levels in MPR mice (*n* = 133, *n* ≥ 11 of each time point (except *n* = 0 at 19, 21 and *n* = 2 at 23 weeks)) and MP mice (*n* = 133, *n* ≥ 11 of each time point (except *n* = 4 at 19, 23 and *n* = 0 at 21 weeks)) and **h** Serum insulin levels in MPR mice (*n* = 123, *n* ≥ 10 of each time point (except *n* = 0 at 19, 21 and *n* = 1 at 23 weeks)) and MP mice (*n* = 68, *n* ≥ 5 of each time point (except *n* = 2 at 19, *n* = 0 at 21 and *n* = 3 at 23 weeks)) over time. MPR *Men1*^*flox/flox*^ *Pten*^*flox/flox*^ RIP-Cre, MP *Men1*^*flox/flox*^ *Pten*^*flox/flox*^, MR *Men1*^*flox/flox*^ RIP-Cre, PR *Pten*^*flox/flox*^ RIP-Cre

We next investigated the effect of the concomitant loss of Pten and Menin in the pancreas, in comparison with the effect of single *Men1* or *Pten* deletion. We evaluated the histopathology of the pancreas for 66 lethargic MPR mice. Fifty-eight mice (88%) developed tumors of variable size and number. From 13 to 22 weeks (*n* = 43), these MPR mice developed multifocal pancreatic tumors that were macroscopically (Fig. 2a) similar to tumors found in MR mice at 35 weeks (11%, *n* = 9) to after 49 weeks (93%, *n* = 27). From 13 to 22 weeks, MR (*n* = 34) and PR (*n* = 46) mice did not show any tumors in the pancreas macroscopically, and evaluation of the histology of pancreas in MR mice (*n* = 21) and PR mice (*n* = 25) did not find any tumors (Fig. 2a and Supplementary Fig. S3A). The protein expression or deletion of Menin and Pten in the islets/tumors of corresponding genotypes was confirmed using IHC staining. IHC staining in MPR pancreatic tumors indicated clonal insulin immunoreactivity, consistent with β-cell neoplasia. Immunoreactivity for the NE markers synaptophysin and chromogranin A indicated that these tumors were PanNETs (Fig. 2b). The Ki 67 index of MPR tumors was between 0.8% and 6.99% (Fig. 2b) and of MR tumors was <2.62% (Supplementary Fig. S3B). By World Health Organization (WHO) classification [1, 3, 4], MPR tumors were G1/G2 PanNETs while MR tumors were G1 PanNETs. The higher Ki 67 index of MPR tumors suggested a higher proliferation rate than that of MR tumors, which may explain early tumor development in MPR mice than that in MR mice. Loss of *Pten* accelerated tumorigenesis in the absence of *Men1* in the pancreas, indicating that *Pten* and *Men1* suppress tumorigenesis of the PanNETs cooperatively in mice. Despite accelerated tumorigenesis, MPR mice maintained WD G1/G2 histology.

To evaluate the temporal appearance and frequency of tumor formation in MPR mice, the histology of their pancreatic islets was evaluated based on H & E and IHC staining of insulin for β-cells and glucagon for α-cells looking at 2-week intervals starting at 3 weeks. Islets from MPR and MP mice showed positive staining for insulin at all time points (Supplementary Fig. S3C). Islets from MP mice were mostly normal with few islets that were mostly round and <0.2 mm in diameter with normal peripheral distribution of α-cells at any age (Fig. 2c). MPR mice showed hyperplastic islets with increasing numbers of small islets and peripheral α-cell distribution starting at 3 weeks. As mice aged, they exhibited increasing size and number of hyperplastic islets, neoplastic islets, and more tumors accompanied by a gradual disappearance of α-cells (Fig. 2c), indicating a multi-step tumor progression. This was further confirmed by quantitatively measuring the ratio of the islets area per pancreas area (Fig. 2d). Histological evaluation in MPR mice showed tumor onset at 7 weeks (8.7%) (Fig. 2e and Table 1), compared to 23 weeks in MR mice [25]. At 15 weeks or later, over 94.7% MPR mice developed PanNETs. Parallel to PanNET formation, MPR mice gradually developed hypoglycemia and increasing serum insulin levels, while MP mice maintained relatively stable blood glucose and serum insulin levels at all ages (Fig. 2g, h). The MPR PanNETs are insulinomas, similar to mouse MR PanNETs.

**Table 1:** PanNET frequency in MPR mouse model

Age of mice (weeks)	# of total mice monitored	Tumor frequency of all mice (%)	# of female mice monitored	Tumor frequency of female mice (%)	# of male mice monitored	Tumor frequency of male mice (%)
3	12	0	6	0	6	0
5	18	0	10	0	8	0
7	23	8.7	13	7.7	10	10
9	23	43.5	14	50	9	33.3
11	22	68.2	16	68.8	6	66.7
13	27	85.2	15	80	12	91.7
15	23	95.7	6	100	17	94.1
17	17	100			17	100
19	2	100	1	100	1	100
23	2	100			2	100

PanNET pancreatic neuroendocrine tumor, MPR *Men1*^*flox/flox*^ *Pten*^*flox/flox*^ RIP-Cre

### Rapamycin treatment resulted in delayed growth of PanNETs and PitNETs

**Fig. 3:**
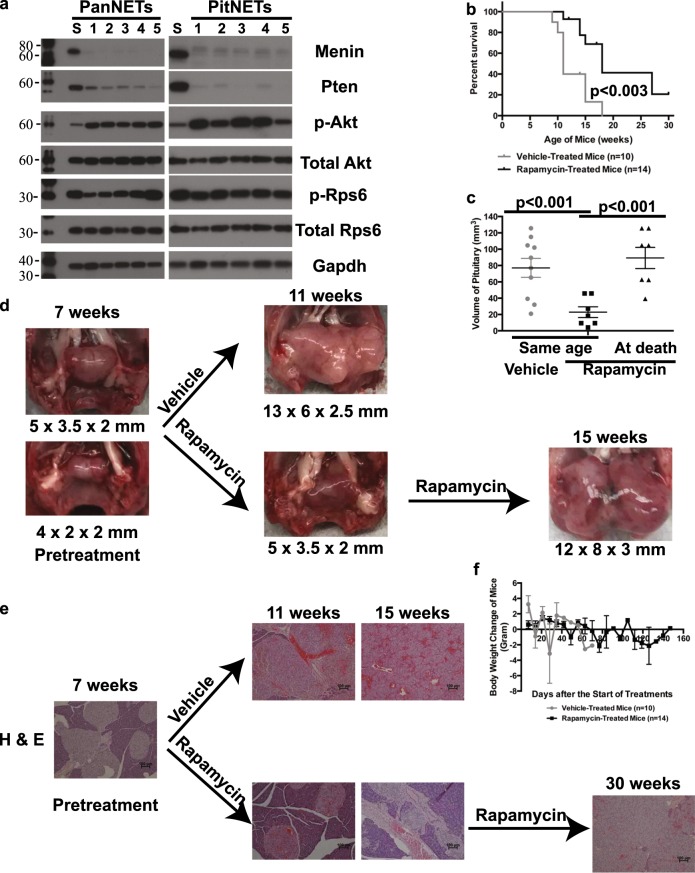
Rapamycin treatments of MPR mice at the onset of tumor delayed growth of pancreatic neuroendocrine tumors (PanNETs) and pituitary neuroendocrine tumors (PitNETs). **a** Western blot analysis of Menin, Pten, phospho-AKT (p-Akt), total Akt, phospho-Rps6 (p-Rps6), total Rps6, and Gapdh proteins from 5 PanNETs and 5 PitNETs of MPR mice shown in Lanes 1–5. Lane S is the spleen from one of the five MPR mice. The molecular weight markers (in kD) were labeled on the left side of the blots. **b**–**d** Rapamycin treatment of MPR mice at the onset of tumor delayed growth of PitNETs and death but did not inhibit PitNET development and death in MPR mice. **b** Rapamycin-treated mice (*n* = 14) showed longer life span than vehicle-treated MPR mice (*n* = 10) (*p* < 0.003) in the first trial. **c** Size of PitNETs of vehicle-treated mice (*n* = 10) was significantly larger than rapamycin-treated mice (*n* = 7) of the same age (*p* < 0.001); size of PitNETs of rapamycin-treated mice (*n* = 7) at death/end of treatment was similar to that of vehicle-treated mice at death (*p* = 0.5). **d** Gross pathology of pituitary in vehicle-treated or rapamycin-treated MPR mice in the first trial. **e** Rapamycin treatments delayed the growth of PanNET in MPR mice—H & E of pancreas in vehicle-treated or rapamycin-treated MPR mice in the first trial. **f** Rapamycin was not toxic to mice. Weekly body weight change of rapamycin- and vehicle-treated mice was shown

To assess signaling alterations in MPR PanNETs and PitNETs, we analyzed the in vivo signaling downstream of Pten and Menin. With Pten loss, Akt, a serine-threonine kinase, is aberrantly phosphorylated and activated in response to PI3K activation. Aberrant activation of mTOR signaling leads to the phosphorylation of downstream effector, ribosomal protein S6 (Rps6). We found increased activation of p-Akt and p-Rps6 in PanNETs and PitNETs in MPR mice (Fig. 3a) as expected with disruption of Pten function.

As a proof of concept and to test the efficacy of our model in preclinical assessments, we set up two trials with the MPR mice using the mTOR inhibitor rapamycin to test for anticancer effects. The first trial tested whether rapamycin treatments could inhibit tumor growth in MPR mice. Treatment started at the onset of tumor development (7–9 weeks). We followed the survival on vehicle-treated mice. When vehicle-treated mice were lethargic, sex-matched, rapamycin-treated littermates (*n* = 7) were sacrificed while other rapamycin-treated mice (*n* = 7) remained in the trial and were sacrificed when six of them were lethargic and one was at 30 weeks to end the treatment. The KMS curve indicated that rapamycin treatments increased the life span of MPR mice (*p* < 0.003; Fig. 3b). Autopsy of these treated mice showed that the pituitaries were significantly smaller in rapamycin-treated mice than in vehicle-treated mice (*p* < 0.001) of the same age (Fig. 3c, d). At death, the pituitaries in rapamycin-treated mice were large and the same size as those of the vehicle-treated mice (*p* = 0.50). The one rapamycin-treated mouse that was alive at 30 weeks had a small PitNET, further supporting that death of MPR mice was due to development of large PitNETs. Similarly, histological examination of the pancreas showed that rapamycin-treated mice exhibited hyperplastic islets while vehicle-treated mice of the same age had PanNETs. Eventually, the rapamycin-treated mice developed PanNETs as well (Fig. 3e). Rapamycin treatment did not show any toxicity based on the measurement of body weight every week (Fig. 3f). Targeting efficacy of rapamycin on these mice was confirmed based on IHC staining of p-Rps6 in these mice (Supplementary Fig. S4E). We also performed a second trial to investigate whether rapamycin treatment could inhibit tumor growth when treatment started before tumor onset (Supplementary section and Supplementary Fig. S4A–D). Collectively, rapamycin treatment delayed the PanNET and PitNET growth but did not inhibit tumor development, and rapamycin treatment increased life span but did not prevent death in MPR mice.

### Another GEMM developed only WD PanNETs

The disadvantage of the RIP-Cre construct is that the Cre expression also occurs in the pituitary due to expression of the Insulin 2 gene in hypothalamus [24]. As MEN1 patients could develop both PitNETs and PanNETs in one person in some cases, this MPR model is not necessarily a disadvantage in terms of understanding the molecular mechanism of the human disease. However, it is difficult to use this model as in vivo preclinical model for PanNETs since both PanNETs and PitNETs develop in the same MPR mouse, and PitNETs are the more lethal tumors. A Cre mouse model with Cre gene expression driven by a knock-in mouse Insulin 1 promoter (MIP-Cre) was recently reported to recapitulate the expression pattern of the endogenous mouse Insulin 1 gene with highly specific targeting to the pancreatic β-cells. MIP-Cre expression does not appear in the brain and other tissues [44].

**Fig. 4:**
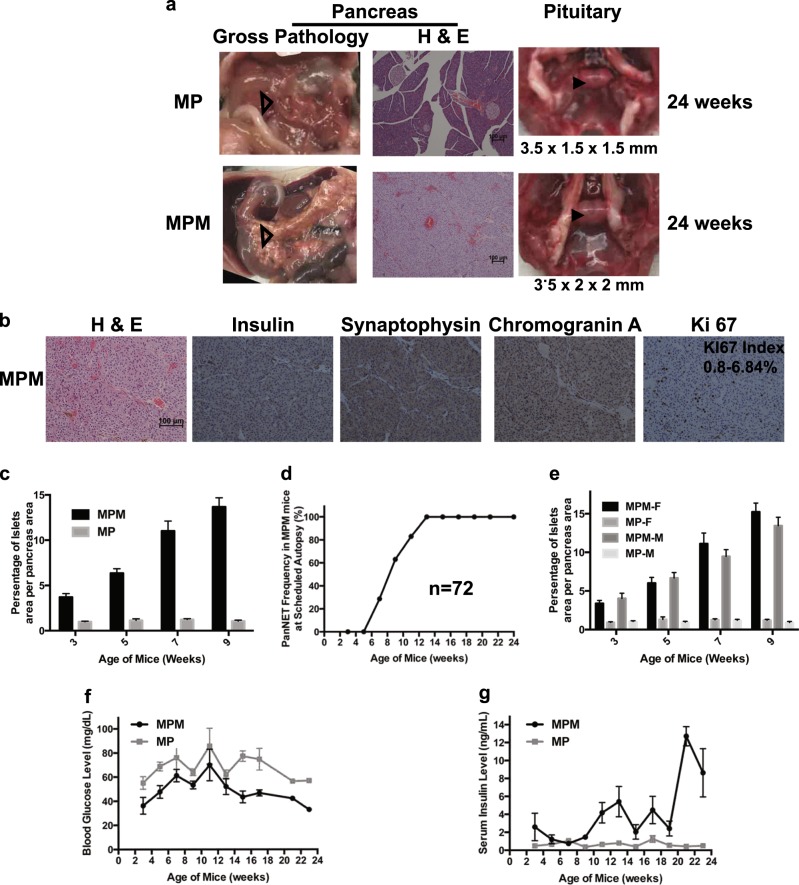
Another well-differentiated pancreatic neuroendocrine tumor (PanNET) mouse model—MPM. **a** Gross pathology of pancreas and pituitary, and hematoxylin and eosin (H & E) staining of pancreas at 24 weeks in MP and MPM mice. Pancreas is shown with open triangle inside the mouse abdomen and pituitary is shown with arrowhead inside the mouse brain skull. **b** MPM tumors were G1/G2 PanNETs—H & E and immunohistochemical staining of insulin, synaptophysin, chromogranin A, and Ki 67 in MPM mice. Ki 67 index (*n* = 16) is shown. **c** Quantitative measurements of the ratio of the islet area per pancreas area in MP and MPM mice as mice aged (*n* ≥ 6 of each time point and each genotype). **d** Tumor frequency in MPM mice (*n* = 72; examined mice at weeks of 3 (*n* = 7), 5 (*n* = 7), 7 (*n* = 7), 9 (*n* = 8), 11 (*n* = 6), 13 (*n* = 10), 15 (*n* = 4), 17 (*n* = 12), 19 (*n* = 3), 21 (*n* = 5), and 23 (*n* = 3). **e** Quantitative measurements of the ratio of the islet area per pancreas area in female and male MP and MPM mice as mice aged (*n* ≥ 3 of each time point, each genotype, and sex). **f** Blood glucose levels in MP and MPM mice as mice aged (*n* = 65 for MPM (*n* = 7, 7, 7, 8, 4, 10, 4, 12, 0, 5, 1 at 3, 5, 7, 9, 11, 13, 15, 17, 19, 21 and 23 weeks, respectively) and *n* = 64 for MP (*n* = 8, 8, 7, 7, 4, 12, 7, 7, 0, 3, 1 at 3, 5, 7, 9, 11, 13, 15, 17, 19, 21 and 23 weeks, respectively)) and **g** serum insulin levels in MP and MPM mice as mice aged (*n* = 68 for MPM (*n* = 6, 7, 7, 8, 5, 9, 4, 12, 3, 5, 2 at 3, 5, 7, 9, 11, 13, 15, 17, 19, 21 and 23 weeks, respectively) and *n* = 65 for MP (*n* = 8, 9, 8, 6, 5, 12, 7, 7, 1, 2, 0 at 3, 5, 7, 9, 11, 13, 15, 17, 19, 21 and 23 weeks, respectively)) MPM *Men1*^*flox/flox*^ *Pten*^*flox/flox*^ MIP-Cre, MP *Men1*^*flox/flox*^ *Pten*^*flox/flox*^, MM *Men1*^*flox/flox*^ MIP-Cre, PM *Pten*^*flox/flox*^ MIP-Cre

We investigated whether conditional deletion of *Men1* and *Pten* using this promoter would also result in PanNETs. Indeed, MPM mice were healthy with a normal pituitary and developed pancreatic tumors at 24 weeks while MP littermates showed normal pituitary and normal pancreas (Fig. 4a). The tumors from MPM mice stained exclusively positive for insulin and NET markers synaptophysin and chromogranin A, indicating that these were PanNETs (Fig. 4b). The Ki 67 index was between 0.8% and 6.84%. Thus the MPM PanNETs were WD G1/G2 PanNETs consistent with MPR PanNETs.

We then evaluated the temporal appearance and frequency of tumor formation in MPM mice with the same criteria and pancreas-related protocols as with MPR mice. Based on H & E and insulin and glucagon immunoreactivities, MPM mice exhibited the same multi-step tumor progression from hyperplastic islets to one tumor to more tumors as observed in the MPR mice (Supplementary Fig. S5A). Quantitatively measuring the ratio of the islets area per pancreas area confirmed that hyperplastic islets appeared at 3 weeks with progressively increasing the ratio of islets area per pancreas area as PanNETs developed while MP mice displayed consistent ratio of islets area per pancreas area at all ages (Fig. 4c). Histological evaluation of the pancreas of the cohort MPM mice indicated that around 28.6% of MPM mice developed PanNETs at 7 weeks and 100% of mice developed PanNETs at 13 weeks and later (Fig. 4d and Table 2). MPM mice developed hypoglycemia and elevated serum insulin levels as they developed PanNETs (Fig. 4f, g), indicating that these PanNETs were insulinomas, similar to MPR PanNETs. Thus the MPM model showed similar characteristics in the pancreas to MPR mice with no effect on the pituitary.

**Table 2:** PanNET frequency in MPM mouse model

Age of mice (weeks)	# of total mice	Tumor frequency in all mice (%)	# of female mice	Tumor frequency in female mice (%)	# of male mice	Tumor frequency in male mice (%)
3	7	0	3	0	4	0
5	7	0	3	0	4	0
7	7	28.6	3	33.3	4	25
9	8	63	5	66.7	3	66.7
11	6	83	3	100	3	66.7
13	10	100	5	100	5	100
15	4	100	3	100	1	100
17	12	100	5	100	7	100
19	3	100	3	100		
21	5	100	3	100	2	100
24	3	100	2	100	1	100

PanNET pancreatic neuroendocrine tumor, MPM *Men1*^*flox/flox*^ *Pten*^*flox/flox*^ MIP-Cre

**Fig. 5:**
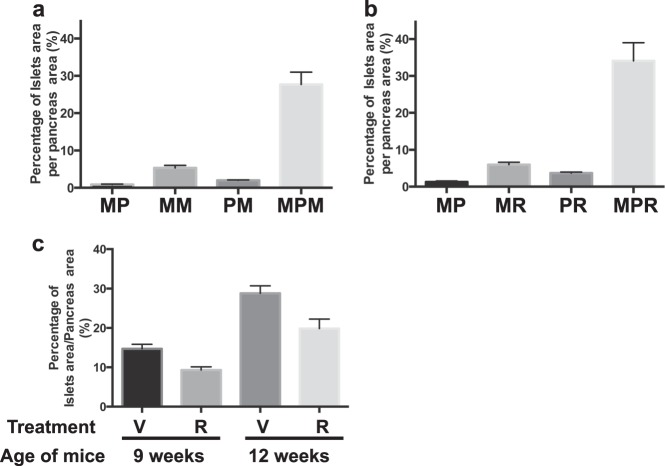
Concomitant loss of *Men1* and *Pten* accelerated pancreatic neuroendocrine tumor development in MPM mice. **a** Quantitative measurements of the ratio of the islet area per pancreas area in MP (*n* = 6), MPM (*n* = 6) mice of 11 weeks, and MM (*n* = 4) and PM (*n* = 6) mice of 18–19 weeks. **b** Quantitative measurements of the ratio of the islets area per pancreas area in MP (*n* = 3), MR (*n* = 3), PR (*n* = 3), and MPR (*n* = 5) mice of 15 weeks. **c** Quantitative measurements of the ratio of the islets area per pancreas area of treated mice in preclinical rapamycin assessment. Vehicle-treated mice (V) at 9 weeks (*n* = 6), Rapamycin-treated mice (R) at 9 weeks (*n* = 5), Vehicle-treated mice (V) at 12 weeks (*n* = 6), Rapamycin-treated mice (R) at 12 weeks (*n* = 8). MPM *Men1*^*flox/flox*^ *Pten*^*flox/flox*^ MIP-Cre, MP *Men1*^*flox/flox*^ *Pten*^*flox/flox*^, MM *Men1*^*flox/flox*^ MIP-Cre, PM *Pten*^*flox/flox*^ MIP-Cre, MPR *Men1*^*flox/flox*^ *Pten*^*flox/flox*^ RIP-Cre, MR *Men1*^*flox/flox*^ RIP-Cre, PR *Pten*^*flox/flox*^ RIP-Cre

To understand whether Menin and Pten function cooperatively to suppress PanNETs in MPM mice, pancreas sections from *Men1*^*flox/flox*^ MIP-Cre (MM) mice at 18 weeks and *Pten*^*flox/flox*^ MIP-Cre (PM) mice at 19 weeks were evaluated histologically. At this age, 100% MPM mice developed PanNETs while MM and PM mice displayed only islet hyperplasia (Supplementary Fig. S5B). MM mice exhibited larger islets and reduced number of α-cells and PM mice exhibited smaller islets with relatively normal distribution of β-cells and α-cells, indicating that MM mice developed more islet abnormalities than PM mice. Quantitative measurements of the ratio of the islets area per pancreas area in the MM and PM mice of 18–19 weeks and in the MP and MPM mice of 11 weeks clearly demonstrated that concomitant loss of *Men1* and *Pten* accelerated PanNET development in MPM mice (Fig. 5a).

To test the efficacy of this MPM model in preclinical assessment, we treated the MPM mice with rapamycin (*n* = 13) and vehicle (*n* = 12) at 4 weeks before the onset of PanNET development. Treatments were ended after 5 weeks in half of the groups of mice and after 8 weeks in the rest of the groups. Histology of the pancreas was evaluated and the ratio of the islets area per pancreas area was quantitatively measured, demonstrating that rapamycin treatments delayed the PanNET growth after 5- or 8-week treatments but did not inhibit PanNET development compared to vehicle-treated littermates (Fig. 5c), as seen in MPR mice. Since MPM mice did not die by 24 weeks, this model provides a better-targeted option for in vivo preclinical therapeutic study for human PanNET patients.

## Discussion

Effective models in preclinical testing are essential in improving clinical outcomes. Motivated by the need for WD PanNET models, we sought tumor suppressors that function cooperatively with Menin to suppress NE tumorigenesis. Using Cre-LoxP system to inactivate *Pten* and *Men1* in β-cells, we generated two mouse models that develop tumors earlier than single deletion of *Men1* or *Pten*: MPR that develops PanNETs and PitNETs in the same mouse, and MPM that develops only PanNETs. Examination of the Ki 67 index of PanNETs indicates that they are WD G1/G2 PanNETs, which are in keeping with the human counterpart PanNETs. The PitNETs developed in MPR mice are prolactinomas, which may be the reason that female mice developed PitNETs faster and larger than male mice. The PanNETs developed in MPR and MPM mice are insulinomas and gender bias was not observed in PanNET development based on the ratio of β-cell mass and tumor development frequency as mice aged (Fig. 2f and Table 1, Fig. 4e and Table 2).

The rapid development of NETs in MPR and MPM mice suggests that Pten and Menin function cooperatively to suppress NE tumorigenesis. The cooperative function of Menin and Pten has not been previously reported in any cancer. Our data are also the first to directly support the importance of the PI3K/AKT/mTOR pathway in NE tumorigenesis in mice. It has been reported that *Pten* deletion does not lead to tumorigenesis in β-cells in mice [42, 45, 46], even with the co-activation of c-Myc. However, our MPR and MPM models demonstrate that *Pten* deletion plays a role in tumorigenesis in β-cells. This suggests that Pten function cooperatively with Menin but not with c-Myc. Our analysis of the ratio of the islets area per pancreas area between MR and PR and between MM and PM (Fig. 5a, b) suggests that Pten plays a less dominant role than Menin in tumorigenesis of β-cells. In addition, our MPR model suggests that Pten deletion plays a role in tumorigenesis of pituitary, which has not been reported before. PR mice showed faster growth of pituitary than MR (Fig. 1e) and displayed PitNETs eventually (Data not shown), suggesting that Pten may play a more dominant role than Menin in tumorigenesis of pituitary. The functional consequence of Menin inactivation is the loss of H3K4me3 on the promoters of Menin-regulated genes, which leads to downregulation of these genes [47]. In endocrine pancreas, Menin-regulated genes are cyclin-dependent kinase inhibitors *p18* and *p27* [48]. Our evaluation on the expression of p27 and p18 in the MPR and MR tumors suggested low or undetected protein expression in MR and MPR tumors (data not shown). Since it has been reported that Pten controls cell cycle by decreasing cyclin D and increasing p27 expression [49], cooperativity of Menin and Pten in NE tumorigenesis may be through regulation of p27. Further investigation of how Menin-mediated and PI3K/AKT/mTOR signaling pathways function cooperatively is worth pursuing.

The Men1 mouse models closely resemble human MEN1 disease but develop PanNETs at a delayed latency, which is not an ideal preclinical model. The RIP-Tag2 mouse model is well characterized with tumor onset at 10 weeks and has proven effective in drug testing for the treatment of advanced PanNETs [12, 50, 51]. However, RIP-Tag2 mice develop high-grade WD G3 PanNETs and PD PanNECs, which are uncommon in human counterpart PanNETs [15]. In addition, the mouse has a T-antigen not found in human and co-mutations of tumor suppressors Rb and p53 have not been reported in human PanNETs. Our MPR and MPM models have the advantages of both Men1 and RIP-Tag2 mouse models. The MPR and MPM models mimic human MEN1-like disease and develop WD G1/G2 PanNETs. Also, co-mutations of *PTEN* and *MEN1* have been found in 8.8% [32] or 13.3% [21] of human PanNET patients with somatic *MEN1* mutations and in 50% [32] or 80% [21] of human PanNET patients with somatic *PTEN* mutations. Like the RIP-Tag2 model, the MPR and MPM models have an earlier onset of PanNETs. Consistent with expectations for such models, the mice were responsive to the well-established rapamycin treatment. Specifically, MPM model develops only PanNETs, allowing preclinical study of drug candidates for WD PanNETs. Our models will complement the RIP-Tag2 mouse model in PanNET therapeutic research [12, 50].

In summary, we demonstrate for the first time that Menin and Pten function cooperatively in suppression of NE tumorigenesis in pancreas and pituitary and have developed two WD PanNET mouse models, which will permit a more detailed exploration of the pathways in NETs. With their similarity to human NETs, these models could prove valuable in preclinical investigation of much needed new therapies for these indolent but progressive and often fatal tumors.

## Supplementary information

supplementary text summary

Supplementary text and supplementary figure legends

Supplementary Figure S1

Supplementary Figure S2

Supplementary Figure S3

Supplementary Figure S4

Supplementary Figure S5
