# Supplementary material for: Two well-differentiated pancreatic neuroendocrine tumor mouse models
Source: Cell Death Differ. 2019 Jun 3;27(1):269–83. doi: 10.1038/s41418-019-0355-0 (PMC7206057; doi:10.1038/s41418-019-0355-0)
Supplement: Supplementary file 1 — supplementary text summary [file 41418_2019_355_MOESM1_ESM.docx]

Supplementary information is about the second preclinical trial we performed on MPR mice treated before the tumor onset, and supplementary Figures S1, S2, S3, S4 and S5.

1. One Word file about the experiment, results and interpretation of second trial of rapamycin treatments, plus Figure legends about the supplementary Figure S1, S2, S3, S4 and S5.
2. Five pdf files of the Supplementary Figure S1, S2, S3, S4 and S5.
