# Supplementary material for: Two well-differentiated pancreatic neuroendocrine tumor mouse models
Source: Cell Death Differ. 2019 Jun 3;27(1):269–83. doi: 10.1038/s41418-019-0355-0 (PMC7206057; doi:10.1038/s41418-019-0355-0)
Supplement: Supplementary file 2 — Supplementary text and supplementary figure legends [file 41418_2019_355_MOESM2_ESM.docx]

**Rapamycin treatment resulted in delayed growth of PanNETs** **in MPR mice**. In the second trial, we investigated whether rapamycin treatment could inhibit tumor growth when treatment started before tumor onset. Rapamycin and vehicle was administered to MPR mice starting at four to five weeks. Rapamycin-treated mice were sacrificed if vehicle-treated littermates showed lethargic or all mice were sacrificed at the end of eight-week treatments. The KMS curve indicated that rapamycin treatments increased the life span of MPR mice (p<0.01) (Supplementary Figure S4A). Five of seven vehicle-treated MPR mice were lethargic due to development of large PitNETs and two vehicle-treated mice with enlarged pituitaries were sacrificed at the end of eight-week treatments. All seven rapamycin-treated mice with elongated pituitaries were healthy at the end of eight-week treatments. The size of pituitaries in rapamycin-treated mice was significantly smaller than that of vehicle-treated mice (p<0.01) (Supplementary Figure S4B). Further, histological evaluation of the pancreas demonstrated that rapamycin-treatment delayed the growth of pancreatic islets. Five of seven vehicle-treated mice developed PanNETs and two developed hyperplastic islets. All seven rapamycin-treated mice showed hyperplastic islets with smaller islets and less abnormal hormone distribution, but the islets were larger compared to the pre-treated mice (Supplementary Figure S4C). Rapamycin treatment did not show any toxicity based on the measurement of body weight every week (Figure S4D). Taken together, rapamycin treatments delayed the growth of PanNETs and PitNETs, and death in MPR mice when treated before tumor onset.

**figure legends**

**Supplementary Figure S1.** Strategy of generating compound mice and results of PCR analysis. (A) Diagram of the strategy used to breed compound mice *Men1^flox/flox^ Pten^flox/flox^* RIP-Cre (MPR) and littermates *Men1^flox/flox^ Pten^flox/flox^* (MP). (B) Representative genotyping results of the litters in (A) by PCR using tail genomic DNA. Genotypes of each lane: 1-WT, 2-RIP-Cre, 3-*Men1^flox/flox^* RIP-Cre (MR), 4-*Pten^flox/flox^* RIP-Cre (PR), 5-*Men1^flox/flox^ Pten^flox/flox^* (MP), 6-*Men1^flox/flox^ Pten^flox/flox^* RIP-Cre (MPR). (C) Tissue-specific deletion of *Men1* and *Pten* in MPR mice was confirmed by PCR using genomic DNA from tissues of various organs, laser capture-microdissected pancreatic exocrine tissues and islets. Tissues of each lane: B-brain, H-heart, I-intestine, K-kidney, LI-liver, Lu- lung, ET-pancreatic exocrine tissue, PI-pancreatic islets, S-spleen.

**Supplementary Figure S2.** Histology of normal pituitary in MP mice – anterior lobe, intermediate lobe (arrowhead) and posterior lobe was shown. MP: *Men1^flox/flox^ Pten^flox/flox^*.

**Supplementary Figure S3.** Concomitant loss of *Men1* and *Pten* accelerated PanNETs. **A.** H & E staining, IHC staining of Menin and Pten on pancreas sections of MP, MPR, MR and PR mice at 15 weeks or 35 weeks**. B.** H & E, and IHC staining of insulin and NET markers on MR pancreatic tumors. Ki 67 index was shown. **C.** IHC staining of insulin of pancreas sections from MPR and MP mice of different ages. MPR: *Men1^flox/flox^ Pten^flox/flox^* RIP-Cre, MP: *Men1^flox/flox^ Pten^flox/flox^*, MR: *Men1^flox/flox^* RIP-Cre, PR: *Pten^flox/flox^* RIP-Cre.

**Supplementary Figure S4.** Rapamycin treatments before the onset of tumor development delayed tumor growth and did not inhibit tumor development in MPR mice. **A.** Kaplan-Meier survival curves of rapamycin-treated (n=7) and vehicle-treated (n=7) MPR mice in the second trial. **B.** Rapamycin-treated mice (n=7) showed significantly smaller pituitaries than vehicle-treated mice (n=7) of the same age (p<0.01). **C.** H & E and IHC glucagon staining of pancreas in vehicle-treated or rapamycin-treated MPR mice in the second trial. **D.** Rapamycin was not toxic to mice. Weekly body weight change of rapamycin-treated and vehicle-treated MPR mice was shown. **E.** Immunohistochemical staining of p-Rps6 on vehicle- and rapamycin-treated MPR mice. MPR: *Men1^flox/flox^ Pten^flox/flox^* RIP-Cre.

**Supplementary Figure S5.** Menin and Pten function cooperatively to suppress tumorigenesis in MPM mouse model. **A.** Multi-step tumor development in MPM mice - H & E, IHC staining of insulin and glucagon of pancreas sections at different ages. **B.** H & E, IHC staining of Insulin, Glucagon, Menin and Pten of MPM, MM and PM mice at 18-19 weeks. MPM: *Men1^flox/flox^ Pten^flox/flox^* MIP-Cre; MP: *Men1^flox/flox^ Pten^flox/flox^*; MM: *Men1^flox/flox^* MIP-Cre; PM: *Pten^flox/flox^* MIP-Cre; MPR: *Men1^flox/flox^ Pten^flox/flox^* RIP-Cre; MR: *Men1^flox/flox^* RIP-Cre; PR: *Pten^flox/flox^* RIP-Cre.
