# Supplementary figures and images for: Two well-differentiated pancreatic neuroendocrine tumor mouse models
Source: Cell Death Differ. 2019 Jun 3;27(1):269–83. doi: 10.1038/s41418-019-0355-0 (PMC7206057; doi:10.1038/s41418-019-0355-0)

H & E

MP

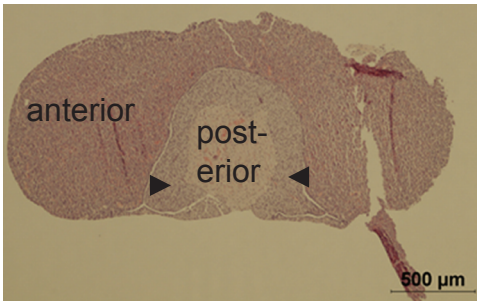

Supplement: Supplementary file 4 — Supplementary Figure S2 [file 41418_2019_355_MOESM4_ESM.pdf]

**A**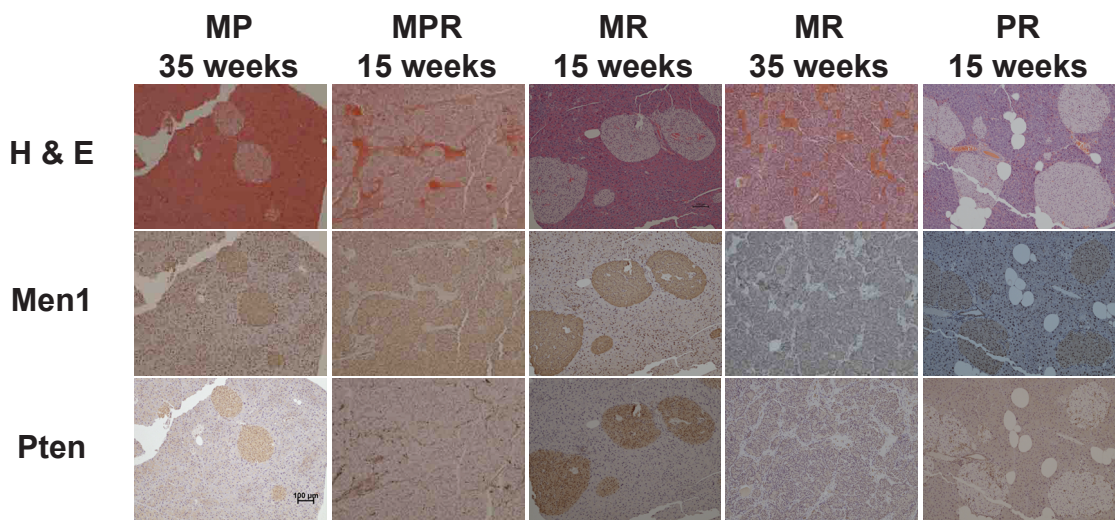**B**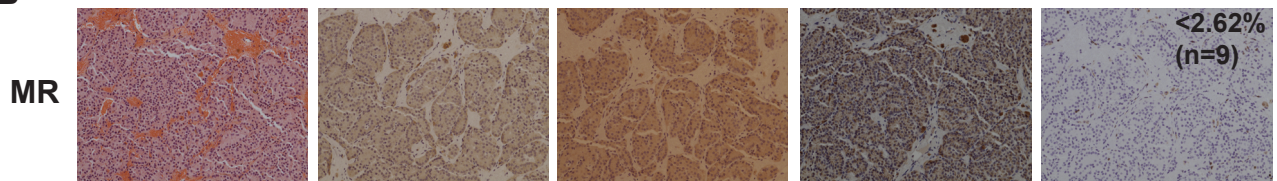**C**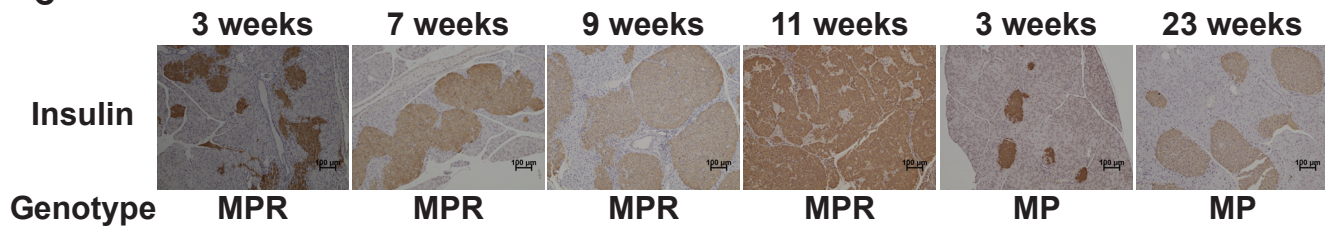

Supplement: Supplementary file 5 — Supplementary Figure S3 [file 41418_2019_355_MOESM5_ESM.pdf]

**A**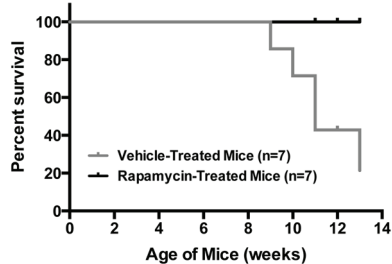**B**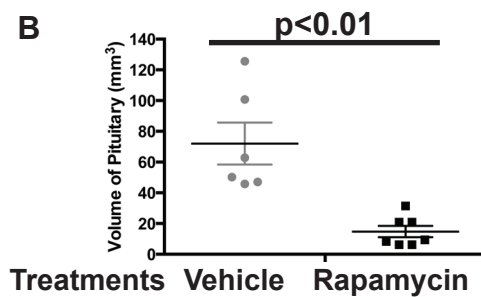**C**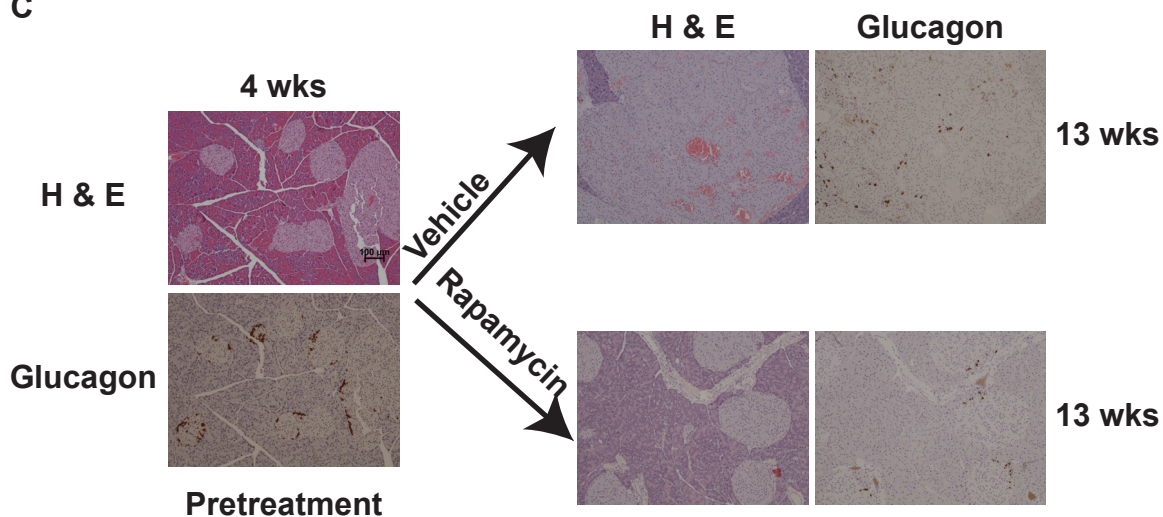**D**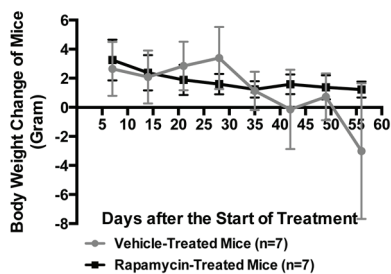**E**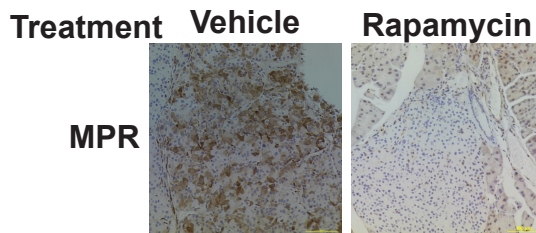

Supplement: Supplementary file 6 — Supplementary Figure S4 [file 41418_2019_355_MOESM6_ESM.pdf]

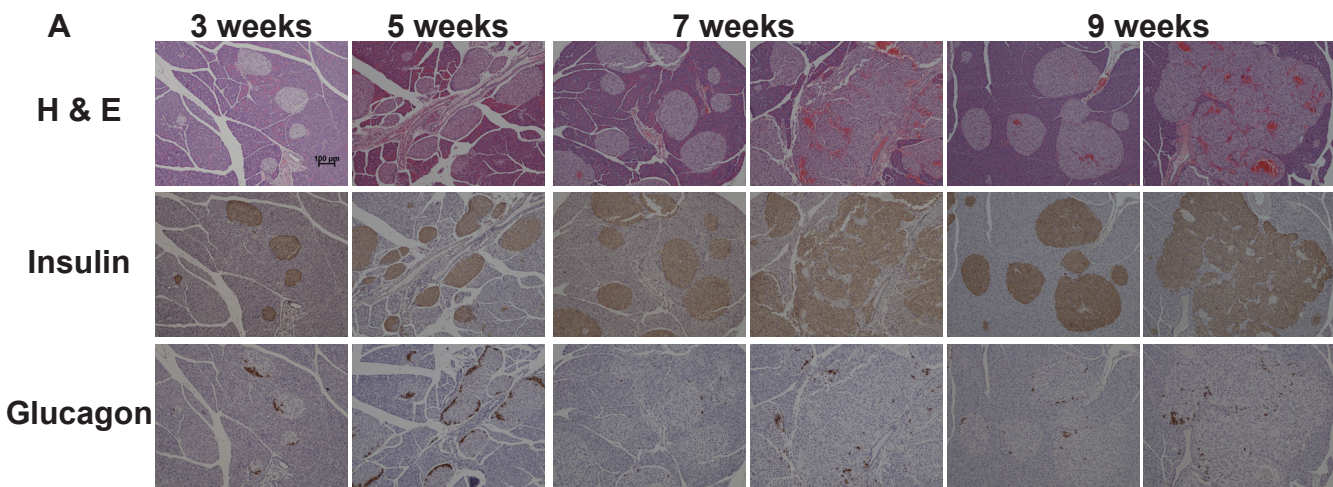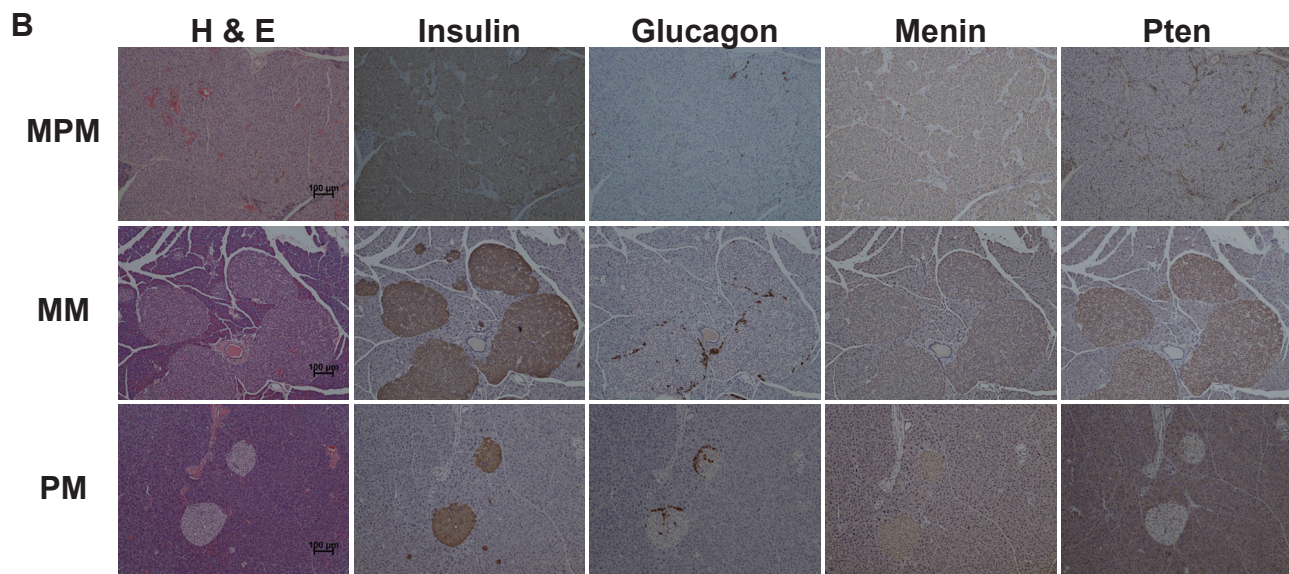

Supplement: Supplementary file 7 — Supplementary Figure S5 [file 41418_2019_355_MOESM7_ESM.pdf]
