# Supplementary material for: Two well-differentiated pancreatic neuroendocrine tumor mouse models
Source: Cell Death Differ. 2019 Jun 3;27(1):269–83. doi: 10.1038/s41418-019-0355-0 (PMC7206057; doi:10.1038/s41418-019-0355-0)
Supplement: Supplementary file 3 — Supplementary Figure S1 [file 41418_2019_355_MOESM3_ESM.pdf]

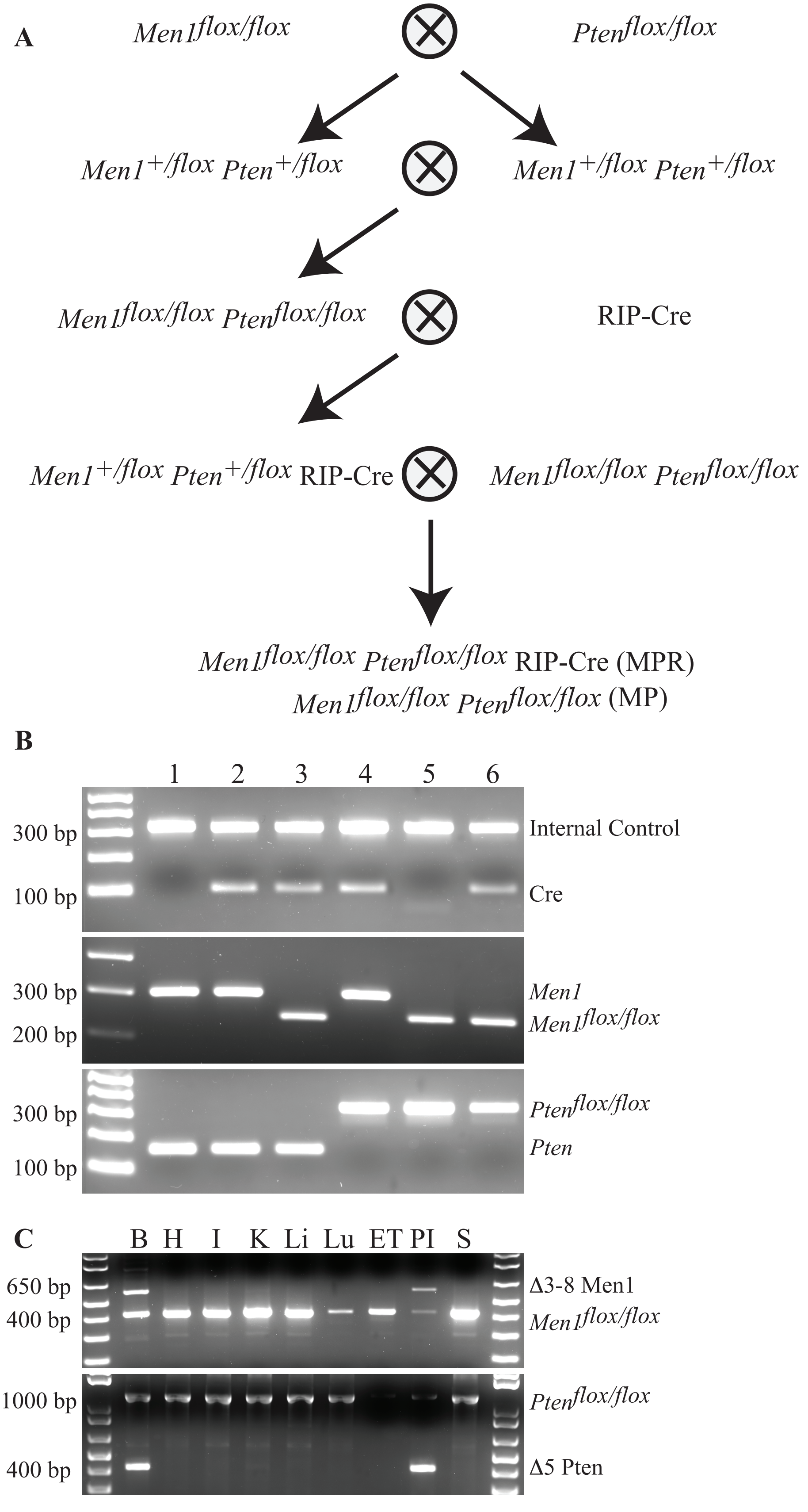
A
Men1flox/flox
Ptenflox/flox
Men1+/flox Pten+/flox
Men1+/flox Pten+/flox
Men1flox/flox Ptenflox/flox
RIP-Cre
Men1+/flox Pten+/flox RIP-Cre
Men1flox/flox Ptenflox/flox
Men1flox/flox Ptenflox/flox RIP-Cre (MPR)
Men1flox/flox Ptenflox/flox (MP)
B
1 2 3 4 5 6
300 bp
100 bp
Internal Control
Cre
300 bp
200 bp
Men1
Men1flox/flox
300 bp
100 bp
Ptenflox/flox
Pten
C
B H I K Li Lu ET PI S
650 bp
400 bp
Δ3-8 Men1
Men1flox/flox
1000 bp
400 bp
Ptenflox/flox
Δ5 Pten
